# Epigenetic and oncogenic regulation of SLC16A7 (MCT2) results in protein over-expression, impacting on signalling and cellular phenotypes in prostate cancer

**DOI:** 10.18632/oncotarget.4328

**Published:** 2015-06-02

**Authors:** Nelma Pertega-Gomes, Jose R. Vizcaino, Sergio Felisbino, Anne Y. Warren, Greg Shaw, Jonathan Kay, Hayley Whitaker, Andy G. Lynch, Lee Fryer, David E. Neal, Charles E. Massie

**Affiliations:** ^1^ Uro-oncology Research Group, CRUK Cambridge Institute, Cambridge, UK; ^2^ Department of Pathology, Centro Hospitalar do Porto, Porto, Portugal; ^3^ Department of Morphology, Institute of Biosciences, Sao Paulo State University (UNESP), Sao Paulo, Brazil; ^4^ Department of Histopathology, Cambridge University Hospitals NHS Foundation Trust, Cambridge, UK; ^5^ Molecular Diagnostics and Therapeutics Group, University College London, London, UK; ^6^ Statistics and Computational Biology Group, CRUK Cambridge Institute, Cambridge, UK; ^7^ Department of Urology, University of Cambridge, Department of Oncology, Addenbrooke's Hospital, Cambridge, UK

**Keywords:** monocarboxylate transporter 2, prostate cancer, castrate resistant disease, malignant phenotype

## Abstract

Monocarboxylate Transporter 2 (MCT2) is a major pyruvate transporter encoded by the SLC16A7 gene. Recent studies pointed to a consistent overexpression of MCT2 in prostate cancer (PCa) suggesting MCT2 as a putative biomarker and molecular target. Despite the importance of this observation the mechanisms involved in MCT2 regulation are unknown. Through an integrative analysis we have discovered that selective demethylation of an internal SLC16A7/MCT2 promoter is a recurrent event in independent PCa cohorts. This demethylation is associated with expression of isoforms differing only in 5′-UTR translational control motifs, providing one contributing mechanism for MCT2 protein overexpression in PCa. Genes co-expressed with SLC16A7/MCT2 also clustered in oncogenic-related pathways and effectors of these signalling pathways were found to bind at the SLC16A7/MCT2 gene locus. Finally, MCT2 knock-down attenuated the growth of PCa cells. The present study unveils an unexpected epigenetic regulation of SLC16A7/MCT2 isoforms and identifies a link between SLC16A7/MCT2, Androgen Receptor (AR), ETS-related gene (ERG) and other oncogenic pathways in PCa. These results underscore the importance of combining data from epigenetic, transcriptomic and protein level changes to allow more comprehensive insights into the mechanisms underlying protein expression, that in our case provide additional weight to MCT2 as a candidate biomarker and molecular target in PCa.

## INTRODUCTION

Prostate cancer (PCa) is the third leading cause of cancer death in developed countries and the most common cancer in men and involves a challenging diagnosis [1]. Recent studies point to a consistent overexpression of Monocarboxylate Transporter 2 (MCT2) in PCa [2, 3], which, in terms of sensitivity and specificity to detect malignant glands was comparable to Alpha-Methyacyl-CoA-Racemase (AMACR) an established prostate cancer biomarker. This raises the possibility that MCT2 could have an important functional role in PCa disease [2]. MCT2 has the highest affinity for pyruvate and lactate of all the functionally characterized MCTs being a primary pyruvate transporter in man [4, 5]. In human cancers MCT2 has been described to be strongly expressed in the cytoplasm of colorectal cancer cells indicating a possible role in intracellular organelles such as mitochondria [6]. Also, MCT2 is the primary isoform expressed in human glioblastoma multiform and glioma-derived cell lines [7]. A more recent study in colorectal cancer described that MCT2 knockdown suppressed KRAS mutant colorectal tumour growth *in vivo,* indicating MCT2 as a promising target in colorectal cancer [8].

Despite the recent observation of MCT2 expression in PCa tumours the mechanisms of over-expression remains unknown and it is not known if this expression is maintained across different stages of the disease. Also, the impact of MCT2 inhibition in PCa cells is still unknown and links between SLC16A7/MCT2 and major prostate cancer drivers such as Androgen Receptor (AR) ETS-related genes (ERG) have not previously been studied. In this study we propose a rationale for the increase of MCT2 expression through an integrative analysis of epigenetic, transcriptome and protein level data from prostate cancer tissue and unveil a link between SLC16A7/MCT2 and major oncogenic pathways in prostate cancer.

## RESULTS

### A selective demethylation at the SCL16A7 locus occurs in PCa compared to benign tissue

**Figure 1:**
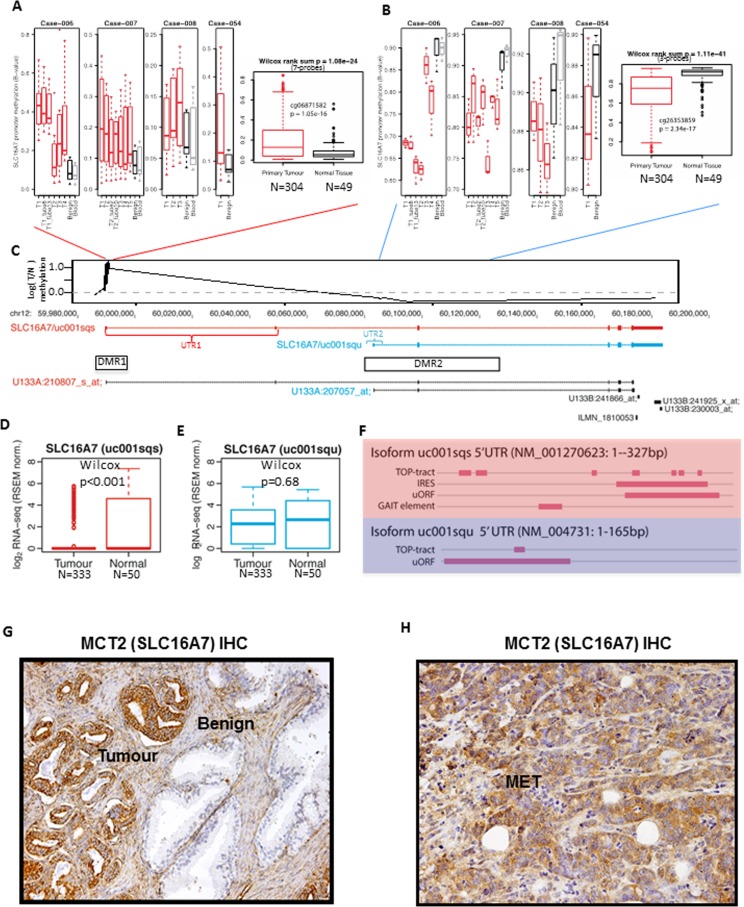
A selective demethylation at the SCL16A7 locus occurs in PCa compared to benign tissue **A.**-**B.** Boxplot summaries of DNA methylation at the SLC16A7/MCT2 gene locus showing results from (left panel) a cohort of prostatectomy specimens with multiple tumour cores (T1-4) and correspondent benign tissue and blood samples and (right panel) a cohort of 304 PCa tumours and 49 benign samples (TCGA). Panel-A depicts data from the main SLC16A7/MCT2 promoter and panel-B depicts data from an alternative internal promoter site. *P-*values denote non-parametric Wilcox Rank Sum tests for each promoter region (above graphs) and for representative probes (probe name and *P-*value shown in graph). **C.** Genome browser view of the SLC16A7/MCT2 locus showing the median tumour/normal methylation ratio for 304 TCGA prostate tumour samples (top, black line), expressed SLC16A7/MCT2 isoforms (top, red and blue), the location of the differentially methylated regions (DMR1 and DMR2) and expression array probe sets (bottom panel). **D.**-**E.** Boxplot showing expression of SLC16A7/MCT2 long **D.** and short **E.** isoforms in PCa tumour and benign tissues (TCGA RNA-seq). *P-*values denote non-parametric Wilcox Rank Sum tests. **F.** Summary of 5′-UTR motif analysis of the otherwise identical long (upper panel) and short (lower panel) isoforms of SLC16A7/MCT2. Showing occurrence and location of internal ribosome entry sites (IRES), gamma-IFN activated inhibitor of translation elements (GAIT), mTOR-regulated terminal oligopyrimidine tracts (TOP) and translation-inhibitor/stress-related upstream open reading frame sequences (uORF). **G.** Immunohistochemical expression of MCT2 (100X) in prostate benign glands and prostate tumour glands. **H.** Immunohistochemical expression of MCT2 (200X) in prostate cancer bone metastasis (MET).

In a cohort of four PCa tumours with matched non-malignant tissue we found two differentially methylated regions (DMRs) at the SLC16A7 locus. At the promoter upstream of the full-length SLC16A7/MCT2 isoform we observed an increase in DNA methylation in prostate tumours (DMR1) and at an internal, alternative promoter for SCL16A7/MCT2 locus (DMR2) we observed recurrent demethylation in PCa compared to benign tissue, both within and between patients (Figure 1A-1B). Analysis of methylation profiling from a large cohort of PCa tumours (*n* = 304) showed that demethylation at the internal SLC16A7/MCT2 promoter region and hypermethylation at the upstream promoter is a recurrent and significant change in PCa tumours (Wilcox test *p* < 0.001; Figure 1A-1B). These differentially methylated regions (DMRs) at the SLC16A7/MCT2 locus mapped to a promoter upstream of full-length SLC16A7/MCT2 (DMR1) and an internal promoter region (DMR2, Figure 1C). RNA-sequencing analysis revealed a switch in SLC16A7/MCT2 isoform expression between benign and PCa tumours, defined by repression of the full-length SLC16A7/MCT2 isoform and maintained expression of an alternative isoform arising from an internal promoter (Figure 1D-1E), consistent with the reciprocal DNA methylation changes observed. These alternative isoforms of SLC16A7/MCT2 contain identical coding sequences and differ only in their 5′-UTR sequences (Figure 1C), analysis of which revealed key differences in motifs governing translational mechanisms between the isoforms expressed in tumour and benign prostate samples (Figure 1F). Together these findings suggest that selective methylation and demethylation occurs in prostate tumours at two distinct promoter regions within the SLC16A7/MCT2 locus, resulting in expression of an alternative isoform containing a different set of 5′-UTR translation signals. This acquired epigenetic change therefore represents one possible mechanism responsible for the robust increase of MCT2 protein expression observed in prostate tumours.

Immunohistochemical staining for MCT2 in human prostate tissue confirmed intense staining in tumour glands and low or absent staining in adjacent benign glands (Figure 1G). Also, 7 out of the 10 PCa bone metastasis analysed were positive for MCT2 expression (Figure 1H), showing for the first time the presence of MCT2 expression in metastatic prostate cancer.

### MCT2 expression persists in hormone-refractory disease and SLC16A7/MCT2 is strongly linked with ERG

**Figure 2:**
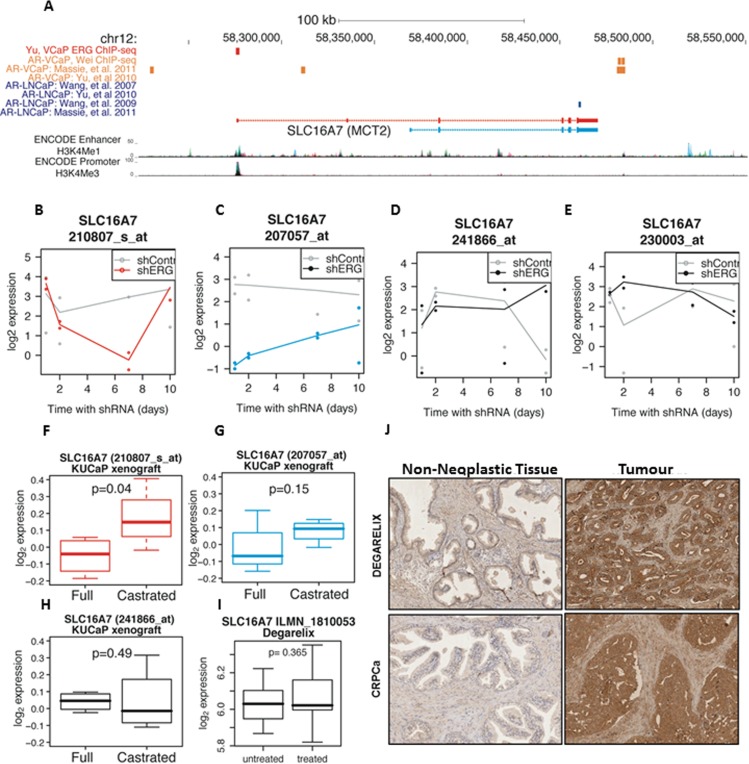
MCT2 expression persists in hormone-refractory disease and SLC16A7/MCT2 expression is linked with the ETS-related gene ERG **A.** Genome browser view of ChI*P-*Sequencing profiles of the AR and ERG transcription factors at the SLC16A7/MCT2 gene locus in LNCaP and VCaP cell lines. Short and long SLC16A7/MCT2 isoforms are shown, with ENCODE promoter and enhancer tracks. **B.**-**E.** Expression of SLC16A7/MCT2 in VCaP cells with and without ERG knock-down (GSE60771), probe-sets are coloured by the detected isoform using the colour scheme shown in Figure 1E. **F.**-**H.** Expression of SLC16A7/MCT2 in KuCaP PCa xenografts from full and castrated mice (GSE21887), again coloured by isoform. **I.** SLC16A7/MCT2 mRNA levels in patients untreated and treated with degarelix. **J.** Immunohistochemical expression of MCT2 in prostate tumours from patients treated with degarelix and in samples from patients with CRPCa (x200).

The AR and its fusion-gene target TMPRSS2-ERG are important regulators of oncogenic pathways in prostate cancer cells [9-11]. We sought to elucidate the potential cross-regulation between MCT2 and AR signalling. We found both AR and ERG binding sites at the SLC16A7/MCT2 locus from ChIP-sequencing of PCa cell lines (Figure 2A). Notably we found evidence of ERG binding at the main SLC16A7/MCT2 promoter in VCaP cells [10] and also a distinct pattern of AR binding at a downstream enhancer in this TMPRSS2-ERG fusion positive PCa cell line (Figure 2A) [11-13]. ERG knock-down affected the expression of both long and short SCL16A7/MCT2 isoforms in VCaP cells, but with different dynamics (Figure 2B-2C) [14]. However ERG depletion showed little effect on signal for SLC16A7/MCT2 3′-UTR sequences suggesting either selective regulation of specific isoforms by ERG or that other factors dominate in the regulation of levels of transcripts containing these 3′-UTR sequences (Figure 2B-2E).

Consistent with this observation of AR binding sites at the SLC16A7/MCT2 locus we also found that castration of PCa xenografts (thereby blocking AR signalling) affects MCT2 transcript levels, particularly for the long SLC16A7 isoform (Figure 2F-2G). However, probes detecting the 3′-UTR showed no change in response to modulation of AR signalling in PCa xenografts (Figure 2H), both in *in vitro* cultured cells (data not shown) or human tumours following AR blockade (Figure 2I), suggesting that AR signalling effects on SLC16A7 expression are either context or isoform dependent. Finally, we observed strong MCT2 protein expression both in samples from patients treated with AR signalling blockade (Degarelix) and samples derived from patients with castrate resistant PCa (CRPCa) (Figure 2J). There was no significant change in MCT2 protein expression between AR blockade (Degarelix) treated and untreated PCa tumours, suggesting that any AR signalling effects on specific SLC16A7/MCT2 isoforms do not impact on total MCT2 protein levels in the context of human PCa tumours. However, as observed in primary tumours, MCT2 protein expression was selectively increased in malignant tissue compared to adjacent non-neoplastic tissue and MCT2 expression persists in drug-resistant tumours (Figure 2J), suggesting that elevated MCT2 expression is maintained throughout PCa development from neoplasia to late stage, drug-resistant tumours (Figure 2J), underscoring MCT2 as a marker for malignant PCa and as a potential target for therapy.

### *In silico* analysis of SLC16A7/MCT2 association with prostate cancer phenotypes

**Figure 3:**
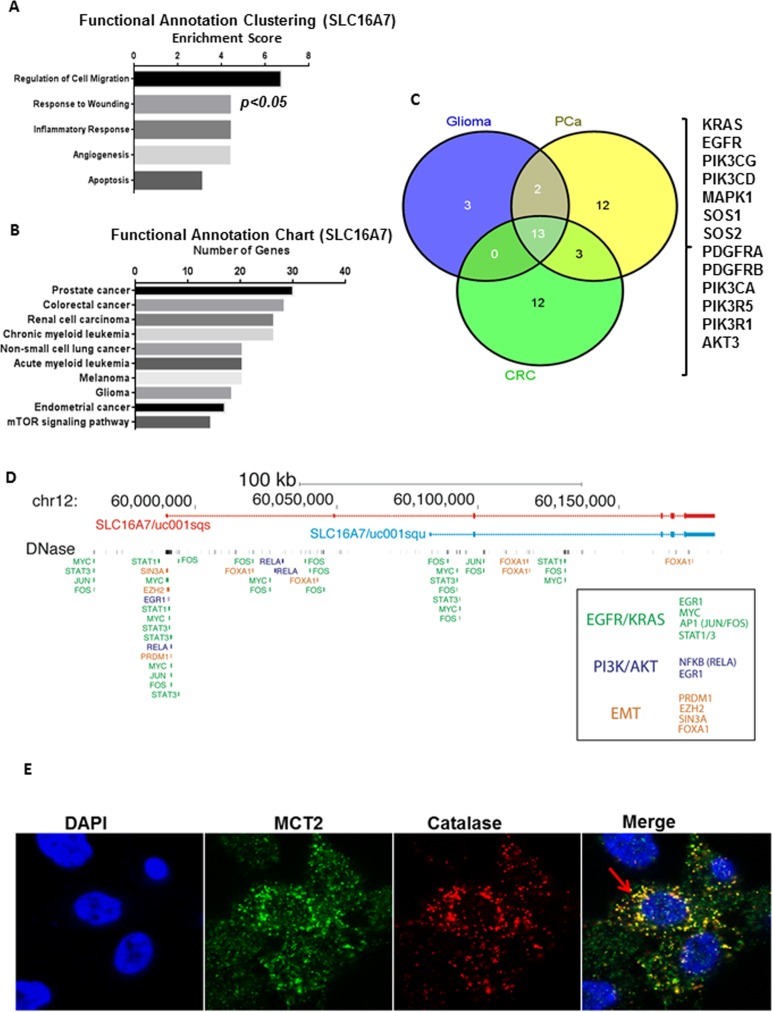
*In silico* validation of the role of SLC16A7/MCT2 expression in PCa aggressive behavior **A.**-**B.** Expression profiles of SLC16A7/MCT2 co-expressed genes were obtained from The Cancer Genome Atlas (cBioportal) and clustered by functional role and signalling pathways using DAVID *in silico* tool. **A.** The panel represents the functional clusters organized by enrichment score and **B.** The panel represents the signalling pathway analysis. **C.** Venn diagram showing the overlap between genes expressed by CRC, glioma and PCa and associated with SLC16A7/MCT2 expression. A list of genes co-expressed with SLC16A7/MCT2 is represented. **D.** Genome browser view of the SLC16A7/MCT2 locus annotated with ENCODE ChI*P-*sequencing data for regulators linked to the signalling molecules found in Fig. 4C. Annotations are coloured by known links to EGFR/KRAS, PI3K/AKT or EMT signalling pathways. **E.** MCT2 co-localizes with catalase at peroxisomes in PCa cells. MCT2 intracellular localization in PC3 cells, DAPI, MCT2, catalase and merged confocal image. Arrows indicate some of the co localization sites.

To further examine the importance of SLC16A7/MCT2 in PCa tumourigenesis we extended our analysis to microarray profiling datasets of PCa tissues available on the cBioportal database [15]. We clustered genes co-expressed with SLC16A7/MCT2 (using probes common to SLC16A7/MCT2 isoforms) in prostate tissues available by their functional role and importance in signal transduction pathways using the DAVID bioinformatics tool (Figure 3A-3B) [16]. We found that the genes co-expressed with SLC16A7/MCT2 were functionally clustered in the categories of regulation of cell migration, response to wounding, inflammatory response, angiogenesis and apoptosis, all considered relevant for tumor progression and development (Figure 3A). A sub-analysis by KEGG signalling pathways revealed that SLC16A7 co-expressed genes are grouped in pathways associated with PCa, colorectal cancer, renal cell carcinoma, leukemia, melanoma, glioma and mTOR signalling pathway among others (Figure 3B). We further explored the common MCT2 correlating genes between glioma, PCa and colorectal carcinoma (CRC), the only tumor types where MCT2 protein expression has been reported (Figure 3C). The core set of 13 genes common to these three tumor types included key regulators of major oncogenic pathways involved in tumor aggressiveness, including KRAS, EGF3 and PI3K (Figure 3C). The correlation of SLC16A7/MCT2 expression with these oncogenic signalling molecules in CRC, glioma and PCa indicates an association between oncogenic pathways and SLC16A7 gene expression. Using publicly available chromatin binding data sets we observed binding of several transcriptional regulators which lie downstream of EGFR, KRAS, PI3K and EMT signalling pathways (Figure 3D), providing tangible leads for future functional studies aiming to decipher the regulators of MCT2 in cancer. Finally, we observed a clear co-localization of MCT2 with Catalase (a well-known peroxisome marker, Figure 3E) confirming that in PCa MCT2 preferentially localizes at peroxisomes rather than mitochondria in prostate cancer cells [17].

### MCT2 knock down affects cells kinetics and is correlated with EMT gene expression in human PCa tumours

**Figure 4:**
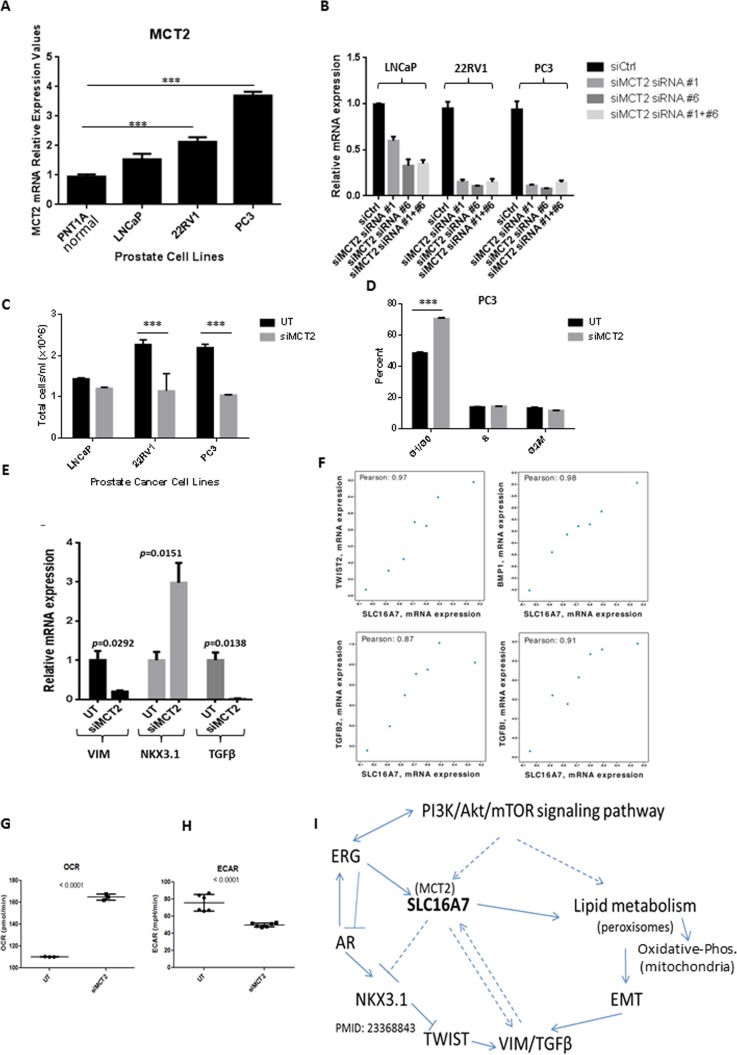
MCT2 knock down affects cells kinetics and is correlated with EMT gene expression in human PCa tumours **A.** mRNA expression levels of MCT2 respectively in prostate-derived cell lines determined by qRT-PCR. Data expressed as mean +/− SD (*n* = 3). (***p < 0.001). **B.** LNCaP, 22RV1 and PC3 cells were transfected with two different combinations of siRNA (siRNA #1 and siRNA #6) directed against MCT2. Successful knock-down was validated using qRT-PCR three days after transfection. Data expressed as mean +/− SD (*n* = 4). **C.**-**E.** Effects of MCT2 knock-down on cell growth. **C.**, Effect of MCT2 knock-down (siMCT2) in a panel of prostate cancer cell lines compared to the untreated cells (UT) after 48h (***p < 0.001). **D.**, Percentage of PC3 cells in G0/G1, S and G2/M fraction when MCT2 is silenced compared to control cells (UT). Data expressed as mean +/− SD (*n* = 3). (***p < 0.001). **E.** To assess effects of MCT2 knock down in genes associated with poor prognosis in PC, PC3 cells were transfected with siRNAs and grown for 3 days. mRNA expression was assessed by qRT-PCR; values are depicted relative to vehicle control for each siRNA. *n* = 4. **F.** Correlation analysis of SLC16A7/MCT2 in PCa tumour cohorts using cBioportal highlighted several EMT-related transcripts with strong correlations (R2 > 0.8) including TWIST2, BMP1, TGFB1 and TGFB2. **G.** Oxygen consumption rates (OCR) and extracellular acidification rates (ECAR) **H.** are shown in control conditions (UT) and when MCT2 (siMCT2) is silenced in PC3 cells. I. Schematic representation of SLC16A7/MCT2 regulation in prostate cancer.

The mRNA levels for MCT2 were readily detectable by qRT-PCR in prostate cell lines compared to immortalized normal prostate epithelial cells (PNT1A) and higher expression was observed at PC3 cells, the highly metastatic and androgen independent model (Figure 4A). To assess the functional importance of MCT2 in PCa cells we used RNAi knock-down and confirmed MCT2 silencing using qRT-PCR three days after transfection (Figure 4B). We observed that MCT2 silencing caused a significant decrease in PCa cell proliferation, particularly for more aggressive PCa cell lines (Figure 4C). A significant increase in the proportion of cells in G0/G1 was detected following MCT2 knock-down in PC3 cells (Figure 4D), indicating that cell cycle arrest was a functional consequence of MCT2 silencing in PCa cells. Additionally, we observed that MCT2 knock-down caused a decrease in the expression levels of Vimentin (VIM) a major player in the epithelial-mesenchymal transition (EMT) process and involved in PCa invasiveness [18]; and transforming growth factor beta (TGFβ), which is associated with metastasis and poor survival in PCa [19]. In contrast the expression of NKX3.1, a prostatic tumor suppressor gene that is down-regulated in a large proportion of high-grade PCa, increased following MCT2 knock-down (Figure 4E). Finally, we found that the expression of several EMT markers correlated tightly with MCT2 expression in PCa tumours (r2 > 0.8; Figure 2F), consistent with our observation of VIM and TGFB depletion in PCa cell lines following MCT2 knock-down and suggesting a link between MCT2 and EMT signalling pathways. Additionally, we observed a significant decrease in extra-cellular acidification rates (ECAR) after MCT2 inhibition and an increase in oxygen consumption rates (OCR) (Figure 4G-4H). These results highlight a non-redundant metabolic role for MCT2 in prostate cancer cells and provide a functional explanation of the clinical relevance of MCT2 over-expression since it is well documented that an increase in extracellular acidification rates is linked with poor prognosis in cancer. In Figure 4I we provide a schematic representation of SLC16A7/MCT2 regulation and down-stream pathways in prostate cancer.

## DISCUSSION

We report a selective demethylation of an internal promoter region within the SLC16A7/MCT2 gene locus and a reciprocal hyper methylation of an upstream promoter region. These methylation changes are associated with differential expression in prostate tumours of an SLC16A7/MCT2 isoform that has an alternative 5′-UTR sequence containing alternative translation control elements. We observed the presence of AR and ERG binding sites at SLC16A7/MCT2 gene locus and found that these oncogenic transcription factors also had differential effects on SLC16A7/MCT2 isoforms with alternative 5′-UTR sequences. The AR and its fusion-gene target ERG also regulate specific isoforms of the gene encoding MCT2 in PCa cells, although the ubiquitous over-expression of MCT2 protein throughout the disease course from primary tumours to CRPC suggests that these transcriptional regulators alone cannot account for the observed MCT2 over-expression in PCa. Importantly, genes co-expressed with SLC16A7/MCT2 in human tumours also clustered in oncogenic-related pathways (e.g. PI3K, EGFR, KRAS) and effectors of these signalling pathways were found to bind at the SLC16A7/MCT2 gene locus (e.g. MYC, EGR1, PRDM1). Therefore, we postulate that a combination of epigenetic changes and oncogenic signalling pathways converge on SLC16A7/MCT2 in prostate cancer cells to effect isoform switching and protein over-expression in PCa tumours. As previously suggested in liver cells [20] it is likely that MCT2 might be involved in a lactate pyruvate shuttle system across the peroxisomal membrane and to form, along with peroxisomal lactate dehydrogenase (pLDH), a peroxisomal lactate shuttle. MCT2 transporters within the peroxisome function to transport pyruvate into the peroxisome where it is reduced by pLDH to lactate. In turn, NADH is converted to NAD+, regenerating this necessary component for subsequent β-oxidation. Lactate is then shuttled out of the peroxisome via MCT2, where it is oxidized by cytoplasmic LDH (cLDH) to pyruvate, generating NADH for energy use and completing the cycle. MCT2 knock-down is likely to disrupt the metabolic balance at peroxisomes with implications for the proliferation rates of PCa cells and the activation of oncogenic pathways in PCa linked with the EMT process. Taken together the peroxisomal localization of MCT2 and the decreased ECAR and increased OCR following MCT2 knock-down suggest that MCT2 inhibition alters flux of pyruvate/lactate into peroxisomes and a metabolic shift to oxidative phosphorylation. Recent studies have established a direct, functional interaction between peroxisomes and mitochondria raising the hypothesis that altered peroxisomal function could directly impact on mitochondria [21], providing one possible mechanism by which MCT2 may be acting in PCa cells.

MCT1 and MCT4 have been extensively described at the plasma membrane of solid tumours as major players in the acid-resistant phenotype of tumour cells [22, 23] and attractive targets for cancer therapy alone or in combination with other drugs targeting metabolism [24], MCT2 appears to contrast with this well-known and stablished function of MCTs 1 and 4 in glycolysis. The strong and cytoplasmic expression of MCT2 in prostate cancer cells not only sheds some light on the involvement of MCT2 in tumours that might not rely mainly on glycolytic metabolism, but also contributes to the understanding of particularities in the metabolism of prostate cancer cells that make it metabolically unique in comparison to other malignancies. These results provide a strong rationale for further studies of the role of MCT2 in PCa and other tumor types, where it may provide new opportunities as either a biomarker for disease tissue or a therapeutic target similar to the recent developments for other members of the monocarboxylate transporter family.

## MATERIALS AND METHODS

### Patient's samples

The present study was previously approved by Local Ethical Review Committee (ref. no. 017/08-010-DEFI/015-CES). For patients exposed to degarelix treatment, informed written consent was received from participants prior to inclusion in the study under ethics committee number MREC 11/H0311/2. Full ethical approval was obtained for all elements of the study including clinical sample collection and analysis NCT01852864 (REC ref:11/H0311/2) and NCT00967889 (REC ref:01/4/061). 27 patients with high-risk PCa (PSA > 20ng/ml or Gleason grade > 7 or clinical stage ≥ cT2c) underwent radical prostatectomy were administered 240mg of degarelix S.C. (donated by Ferring pharmaceuticals) 7 days before undergoing radical prostatectomy. 10 metastatic PCa cases were obtained from clinical biopsy samples.

### Immunohistochemistry (IHC) staining and analysis

IHC technique was performed according to the avidin biotin peroxidase complex principle (R.T.U Vectastain Elite ABC Kit [Universal], Vector Laboratories, Burlingame, CA), with the primary antibodies for MCT2 (1:200, sc-14926, Santa Cruz). MCT2 expression, was analysed in prostate tissue samples using a combined score system. The evaluation was performed blindly by two independent observers that assessed the intensity and the extension of the staining as previously described [2, 3].

### Confocal microscopy

For the immunofluorescence experiments the following antibodies were used: MCT2 (sc 14926, Santa Cruz Biotechnology), and catalase (ab88650, Abcam), and Alexa fluor 488, Alexa fluor 555 secondary antibodies (Invitrogen, Life Technologies).

Immunofluorescence analyses were performed in cells seeded in 8 well Ibidi slides (Thistle Scientific) fixed with 4 % paraformaldehyde in PBS, pH 7.4 for 20 min. Afterwards, cells were permeabilized with 0.2 % Triton X 100 for 10 min, blocked with 1 % BSA solution for 10 min and incubated with primary (MCT2 and catalase) and respective secondary antibodies for 1 h each. Cells were washed in PBS, incubated 10 minutes with Hoescht 33342 stain washed again and digital images recorded using a Leica TCS SP5 confocal microscope Leica, Germany).

### siRNA transfection

siRNA for MCT2 was purchased from Qiagen (Hs_SLC16A7_1, siRNA SI00720531 and Hs_SLC16A7_6, siRNA SI04191348) and transfected using Lipofectamine RNAiMAX (Invitrogen) according to the manufacturer's reverse transfection protocol. Unless otherwise indicated, final siRNA concentrations of 10 nM were used.

### Statistical analysis

Data from human tissue samples were analysed with SPSS statistical software (version 18.0; SPSS) using the Pearson's χ² test, with the threshold for significance being p ≤ 0.05. For *in vitro* studies, GraphPad prism 5 software was used, with the Student's t-test, considering significant values to be p ≤ 0.05.

### *In silico* analysis

Illumina 450k DNA methylation profiling data were retrieved from the ArrayExpress portal (accession E-MTAB-2964) and TCGA data-portal (PCa tumour and benign data). Expression data were retrieved from the TCGA data-portal (PCa tumour and benign RNA-seq isoform level data) or from the Gene Expression Omnibus (GEO, accessions: GSE60771, GSE21887). Chomatin immunoprecipitation data were retrieved from publications (as cited) and ENCODE data were accessed through the UCSC Genome Browser. Data processing and analysis were performed using the R-statistical software and Bioconductor packages [25].
